# Genetic diversity of PRRSV 1 in Central Eastern Europe in 1994–2014: origin and evolution of the virus in the region

**DOI:** 10.1038/s41598-018-26036-w

**Published:** 2018-05-17

**Authors:** Gyula Balka, Katarzyna Podgórska, Manreetpal Singh Brar, Ádám Bálint, Daniel Cadar, Vladimir Celer, Lilla Dénes, Zuzana Dirbakova, Anna Jedryczko, Lázár Márton, Dinko Novosel, Tamaš Petrović, Ivo Sirakov, Dóra Szalay, Ivan Toplak, Frederick Chi-Ching Leung, Tomasz Stadejek

**Affiliations:** 1 Department of Pathology, University of Veterinary Medicine, István Str. 2, H-1076 Budapest, Hungary; 2 National Veterinary Research Institute, Partyzantów 57, 24-100 Pulawy, Poland; 3 The University of Hong Kong, 5N-12 Kadoorie Biological Science Building, Hong Kong, China; 4 National Food Chain Safety Office Veterinary Diagnostic Directorate, Tábornok Str. 2, H-1149 Budapest, Hungary; 5 Arbovirology, Bernhard Nocht Institute for Tropical Medicine, WHO Collaborating Centre for Arbovirus and Haemorrhagic Fever Reference and Research National Reference Centre for Tropical Infectious Diseases, Bernhard Nocht Strasse 74, 20359 Hamburg, Germany; 6 Faculty of Veterinary Medicine, Institute of Infectious Diseases and Microbiology, University of Veterinary and Pharmaceutical Sciences Brno, 612 42 Brno, Czech Republic; 7 Veterinary Institute Zvolen, Pod Drahami 918, 960 86 Zvolen, Slovak Republic; 8 Veterinary Diagnostic Laboratory, Ostródzka, 11-036 Gietrzwałd, Poland; 9 National Food Chain Safety Office Animal Health and Animal Welfare Directorate, Keleti Károly Str. 24, H-1024 Budapest, Hungary; 10 University of Zagreb, Faculty of Agriculture, Department of Animal Science, Svetošimunska cesta 25, 10000 Zagreb, Croatia; 11 Scientific Veterinary Institute ‘‘Novi Sad” Rumenački put 20, 21000 Novi Sad, Serbia; 12 Department of Medical Microbiology, Faculty of Medicine, Medical University of Sofia, Zdrave Str. 2, 1431 Sofia, Bulgaria; 13 University of Ljubljana, Veterinary Faculty, National Veterinary Institute Gerbičeva, 60, 1000 Ljubljana, Slovenia; 14 Department of Pathology and Veterinary Diagnostics, Faculty of Veterinary Medicine, Warsaw University of Life Sciences – SGGW, ul. Nowoursynowska 159c, 02-776 Warsaw, Poland

## Abstract

More than 20 years after the first outbreaks, the phylogenetic picture of PRRSV is still incomplete and full of gaps, especially in regards of PRRSV 1. Due to the exceptional diversity observed at the eastern borders of Europe and the low number of available sequences from Central Eastern European countries, the authors collected and analyzed both recent as well as already submitted sequences comparing them to a large backbone set of available ORF5 sequences representing the full spectrum of PRRSV 1 Subtype 1 diversity to conduct a systematic phylogenetic analysis and reclassification elucidating the diversity of the virus in these countries. Moreover, further analyses of the EUROSTAT data regarding the live pig movement trends revealed their influence of virus diversity and evolution. The results indicate that besides the effect of local, isolated divergent evolution and the use of modified live vaccines, the most important factor influencing a given country’s virus diversity is the transboundary movement of live, infected animals.

## Introduction

After more than 20 years of its discovery, porcine reproductive and respiratory syndrome (PRRS) remains one of the most widespread and economically devastating diseases in swine industry^1,2^. The outbreaks caused by the virus are characterized by reproductive losses in sow herds and respiratory symptoms, as well as increased mortality in young pigs^3^. The disease was first described in the USA, and almost simultaneously, similar symptoms have been observed in Germany^4,5^.

PRRS virus (PRRSV) is a member of the *Porarterivirus* genus of the *Arteriviridae* family within the *Nidovirales* order^6–9^. The relatively small, enveloped virus contains a positive-sense single-stranded RNA genome of approximately 15.1 kb in length encoding 10 ORFs^10–13^.

The viruses found on the two continents caused similar outbreaks, clinical signs and pathological lesions, but soon after their first isolation, marked genetic differences were identified between these strains which were classified into two distinct genotypes (Type 1, formerly EU and Type 2, formerly NA)^7,14^. Moreover, the latest reclassifications suggest the separation of two corresponding species: PRRSV 1 and PRRSV 2, respectively^8,9^.

Initially PRRSV 1 strains were thought to be genetically less variable, however phylogenetic studies performed on Lithuanian, Belarussian and Russian strains revealed an unexpectedly high degree of variability within this genotype and led to the definition of four genetic subtypes^15–18^.

In the recent years, extensive genetic information has been published on PRRSV 1 diversity in Western and Eastern Europe^18–21^. In a detailed phylogenetic study Shi *et al*.^22^ divided the Western European Subtype 1 strains into 12 clades based on inter-clade genetic distances. Information about the virus from Central Eastern Europe however is relatively scarce and has never been analyzed in a systematic way. As some of the Central Eastern European (CEE) countries were members of the political and economic system under Union of Soviet Socialist Republic (USSR) domination, it can be speculated that the Eastern European subtypes of PRRSV 1 might also circulate in the countries of Central Eastern Europe, and not only in the countries formerly being republics of the USSR. The first retrospective evidence of PRRSV in Europe comes from sera obtained in 1987 in the German Democratic Republic, but no genetic data was obtained from those samples^23^. In 1999 a divergent PRRSV 1 strain was detected in Germany^24^, that later was classified in Subtype 2 based on amino acid sequence analysis (Stadejek, unpublished). However, none of the studies confirmed the presence of those subtypes and only Subtype 1 strains among the PRRSV 1 strains were identified. A study published in 2008 revealed that Hungarian PRRSV 1 strains belonged to multiple subgroups within Subtype 1, indicating multiple introductions of PRRS in this country^25^. In Poland similar situation was described^15^. In Slovenia a recent study based on ORF7 phylogeny found relatively high degree of variability among their Subtype 1 strains affecting the sensitivity of several commercial PRRSV detection kits. The authors also suggested multiple introductions of PRRS in their country^26^. A recent study involving relatively low number of sequences revealed that Romanian PRRSV sequences are clustered in three groups within Subtype 1^27^. Another recent paper describing the analysis of PRRSV strains from Slovakia, Czech Republic and a few from Austria revealed that Slovakian strains were clustered into three phylogenetic branches, whereas the Czech and the Austrian ones were mostly forming single nodes on the Subtype 1 part of the tree^28^.

Political changes in Central Eastern Europe in the beginning of the 1990s opened the region to Western Europe and that also influenced animal production. Most of the countries of Central Europe are now members of the EU and in the recent years the systems of pig production have changed and the import of live pigs (finishing and breeding) from other member states has increased. Together with the fast rate of PRRSV evolution, this contributed to a dynamic landscape of virus diversity which was never analyzed in a systematic way.

The aim of this study was to assess PRRSV 1 diversity in Central Eastern Europe and shedding light on the origin and history of the virus in the region.

## Results

### Genetic lineages of PRRSV 1

**Figure 1:**
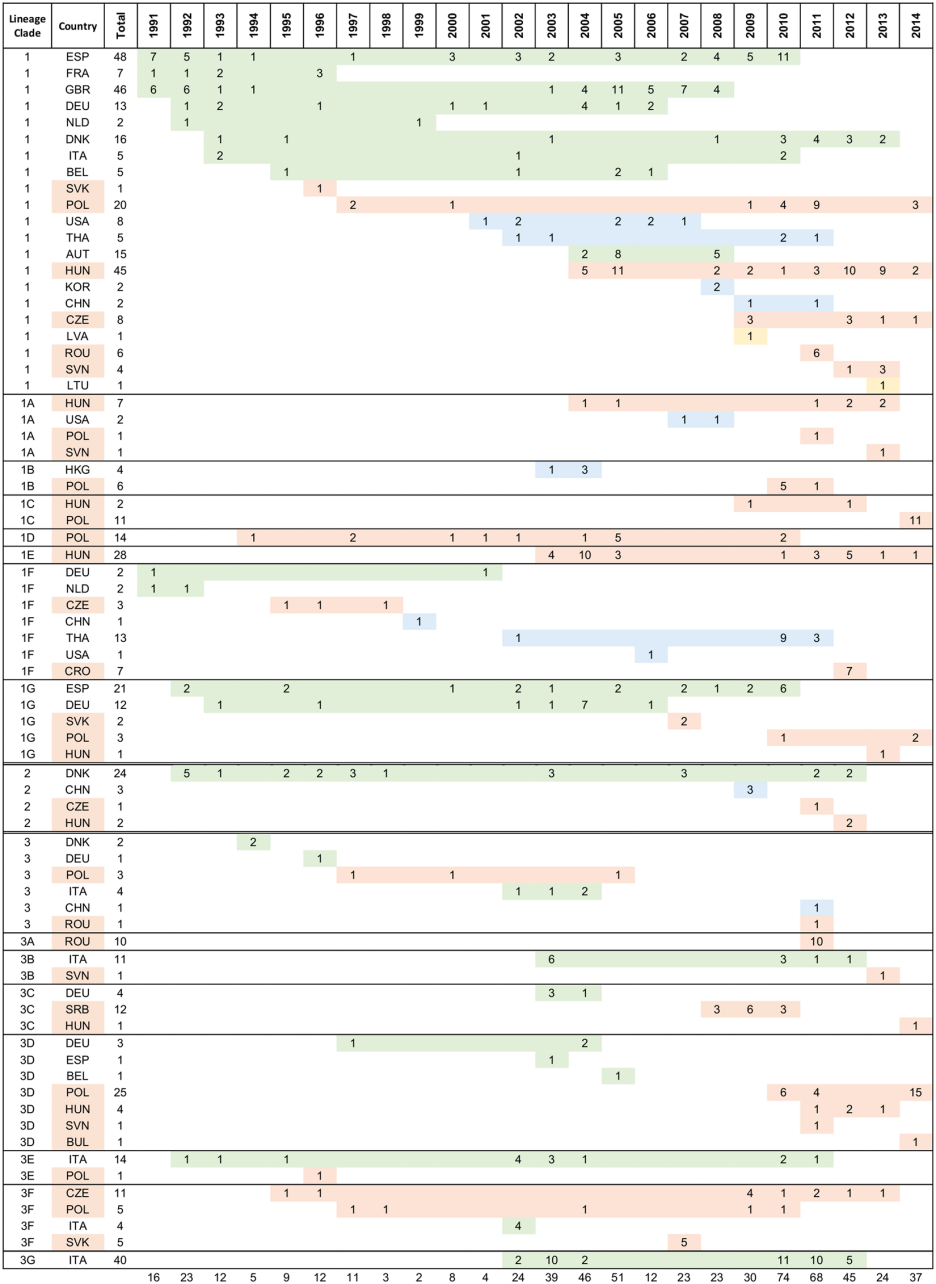
Summary results of phylogenetic analysis (green – Western European countries, orange – CEE countries, blue – outside of Europe, yellow – EE countries).

**Figure 2:**
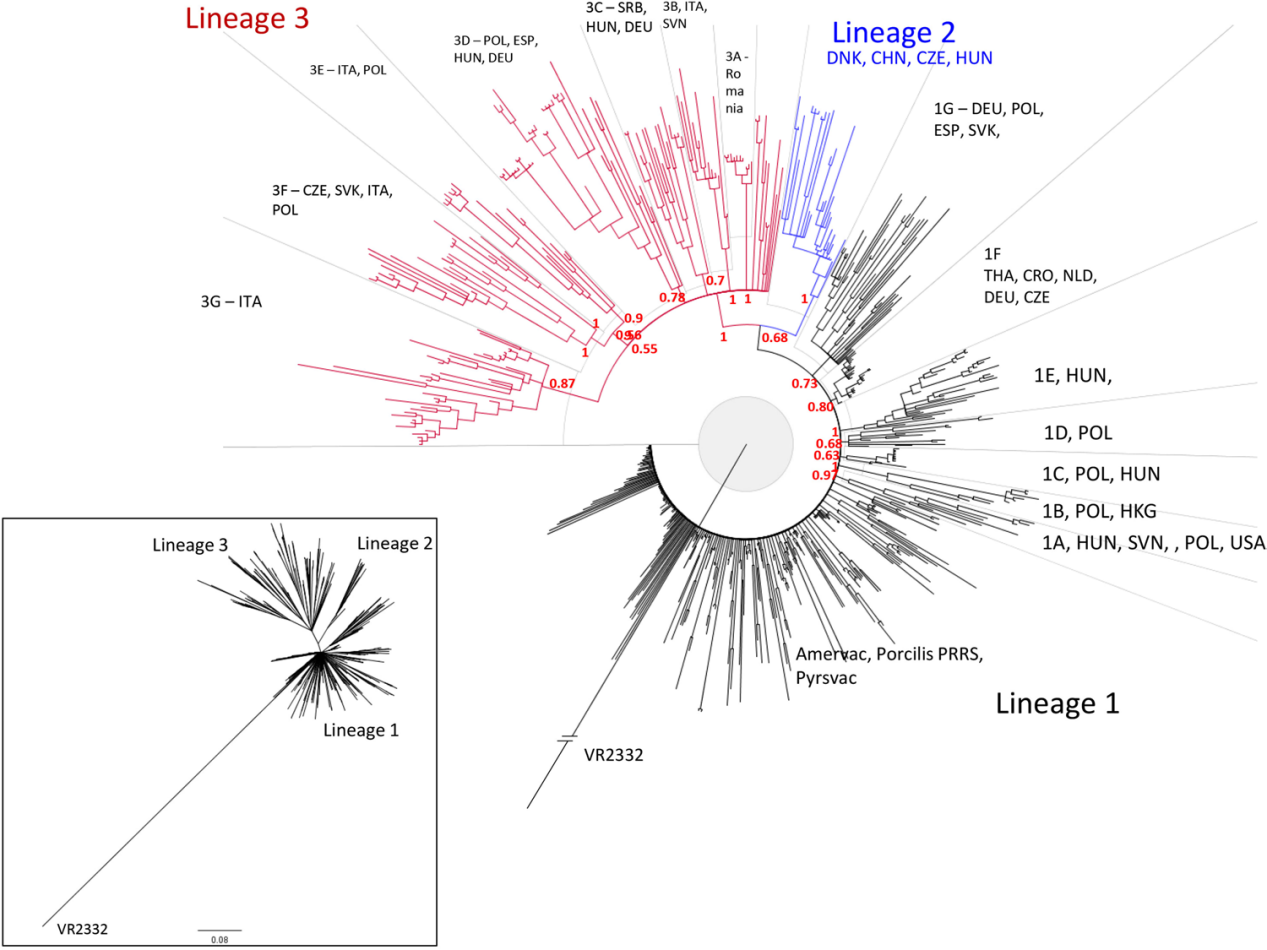
Phylogenetic tree of PRRSV 1 Subtype 1 ORF5 sequences constructed in MrBayes^40^. PRRSV 2 prototype strain VR2332 (AY150564) was used as an outgroup. Three main genetic lineages were distinguished, lineages 1 and 3 containing further clades 1A–1G and 3A–3G, respectively. General geographical distribution was indicated for main clades and lineage 2. Posterior probability values supporting main nodes were shown.

The sequence analyses and subsequent phylogenetic investigations revealed that all Central Eastern European PRRSV 1 strains investigated in this study belonged to three lineages within the genetic Subtype 1 (Figs 1 and 2).

Geographically and structurally diverse *lineage 1* contained 402 sequences from all around the world. These included the PRRSV 1 prototype strain Lelystad as well as Porcilis MLV (MSD Animal Health), Pyrsvac (Syva) and Amervac (Hipra) vaccine strains and possibly field strains originating from them. Interestingly, some strains with significant (97%) pairwise ORF5 nucleotide identity to these vaccines were present in Spain and in Poland in the early 1990s (1994), years before the corresponding vaccine (Amervac) was registered on the market (2002).

*Lineage 1* comprises the oldest Type 1 PRRSV strains collected in 1991 in Spain, France, Great Britain, Germany and the Netherlands. From 1994, when the first strain was identified in Poland, the number of strains from this lineage gradually increased in the CEE countries, reaching the overall number of 167. In Poland it is the most common lineage containing 55 (61.8%) of all Polish sequences from 1994–2014. The second CEE country where lineage 1 strains were detected was Czech Republic, with 11 (47.8%) strains collected between 1995 and 2014. One sequence from 1996 and two sequences from 2007 (37.5%) originated from Slovakia. Lineage 1 also included 6 Romanian (35.5%, 2011), 5 Slovenian (71.4%, 2012–2013) and all 7 Croatian (2012) strains. Lineage 1 was also identified as the most common one in Hungary (83 sequences, 92.2% of all Hungarian sequences), but the oldest strains originated only from 2003. In our study, no lineage 1 sequences were obtained from Serbia and Bulgaria.

**Figure 3:**
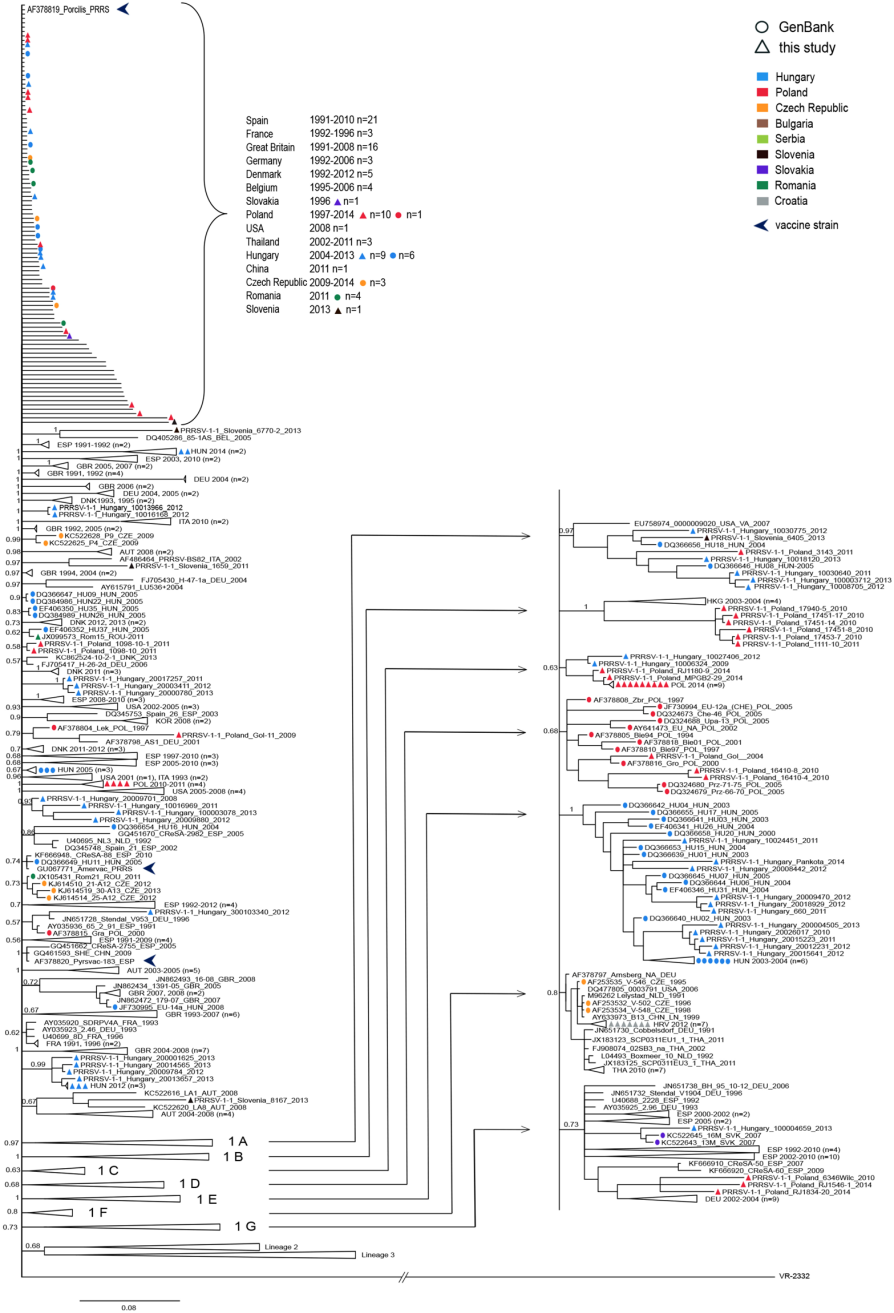
Phylogenetic tree of lineage 1 based on ORF5 sequences constructed in MrBayes^40^. PRRSV 2 prototype strain VR2332 (AY150564) was used as an outgroup. Some minor clusters were collapsed for clarity. Geographical origin and year of isolation were indicated for each sequence. Details of sequences from 1A–1 G clades were shown in the side panel. Posterior probability values supporting main nodes were shown.

Several clades (1 A–1 G) supported by posterior probability values 0.63–1.0 and including 10 or more sequences were distinguished within the lineage 1 (Figs 1, 2 and 3).

Clade 1 A included sequences from Hungary (with the oldest sequence of this clade from 2004) single sequences from Poland and Slovenia and two sequences from USA.

Strains grouped in clade 1B emerged in Hong-Kong in 2003–2004 and in Poland in 2010–2011.

Clade 1 C contained both Hungarian (2009–2012) and Polish (2014) sequences, while clades 1 D and 1 E included solely Polish or Hungarian sequences, respectively.

The oldest sequences from clade 1 F originated from Germany from 1991 and the Netherlands (1991–1992). Between 1995 and 1998 strains of this clade were identified in Czech Republic. Later it spread to Asia and USA. Relatively recently (2012) seven sequences were also detected in Croatia.

Continuous circulation of strains from clade 1 G was observed in Spain and Germany since 1992 and 1993, respectively. Since 2007 strains from clade 1 G spread also to CEE countries - Slovakia, Poland and Hungary.

**Figure 4:**
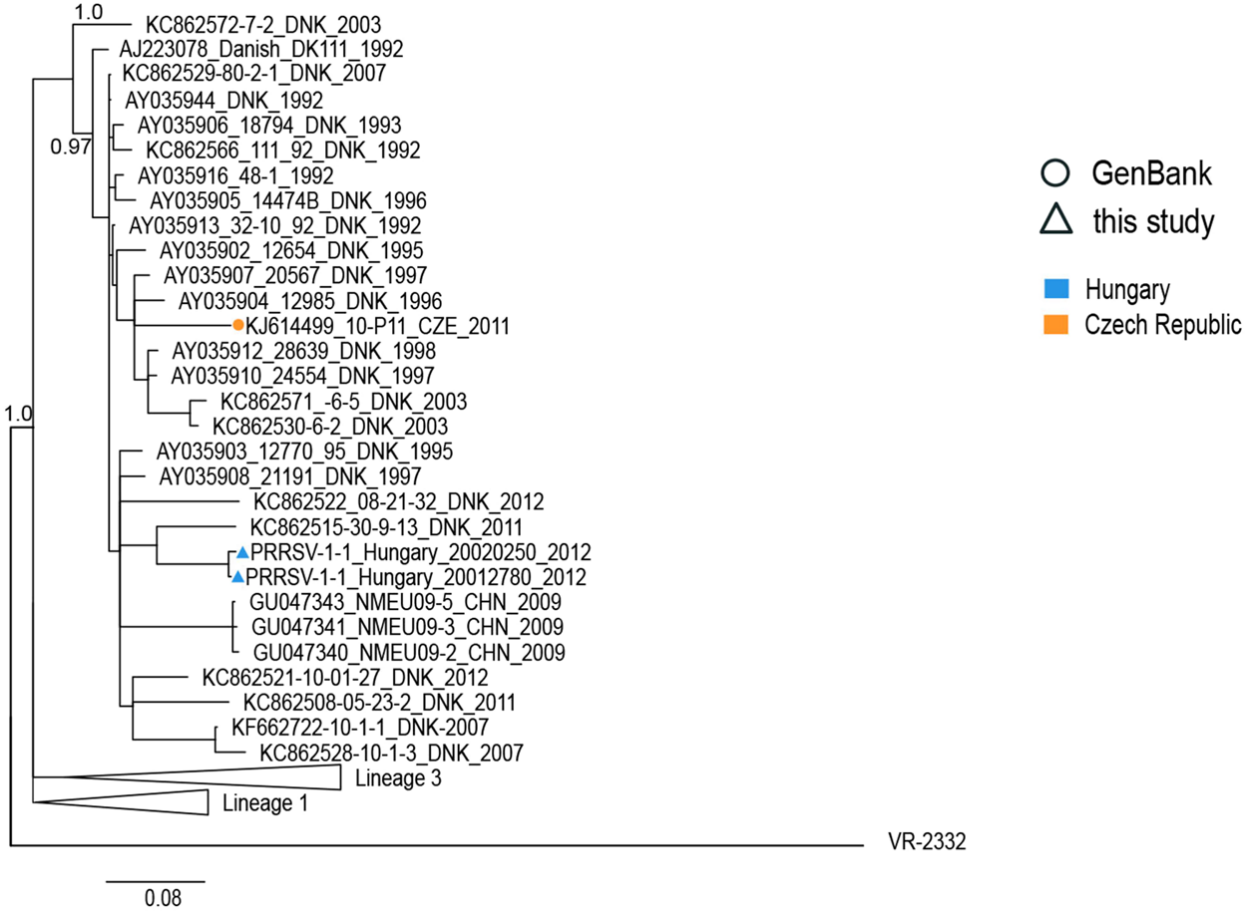
Phylogenetic tree of lineage 2 based on ORF5 sequences constructed in MrBayes^40^. PRRSV 2 prototype strain VR2332 (AY150564) was used as an outgroup. Posterior probability values supporting main nodes were shown. Geographical origin and year of isolation were indicated for each sequence.

*Lineage 2* contained mostly Danish sequences (1992–2012) but also 3 sequences from China (2009), 1 sequence from Czech Republic (2011) and 2 sequences from Hungary (2012) (Figs 1, 2 and 4). According to our data, recent introduction from Denmark is the most plausible origin of these sequences.

**Figure 5:**
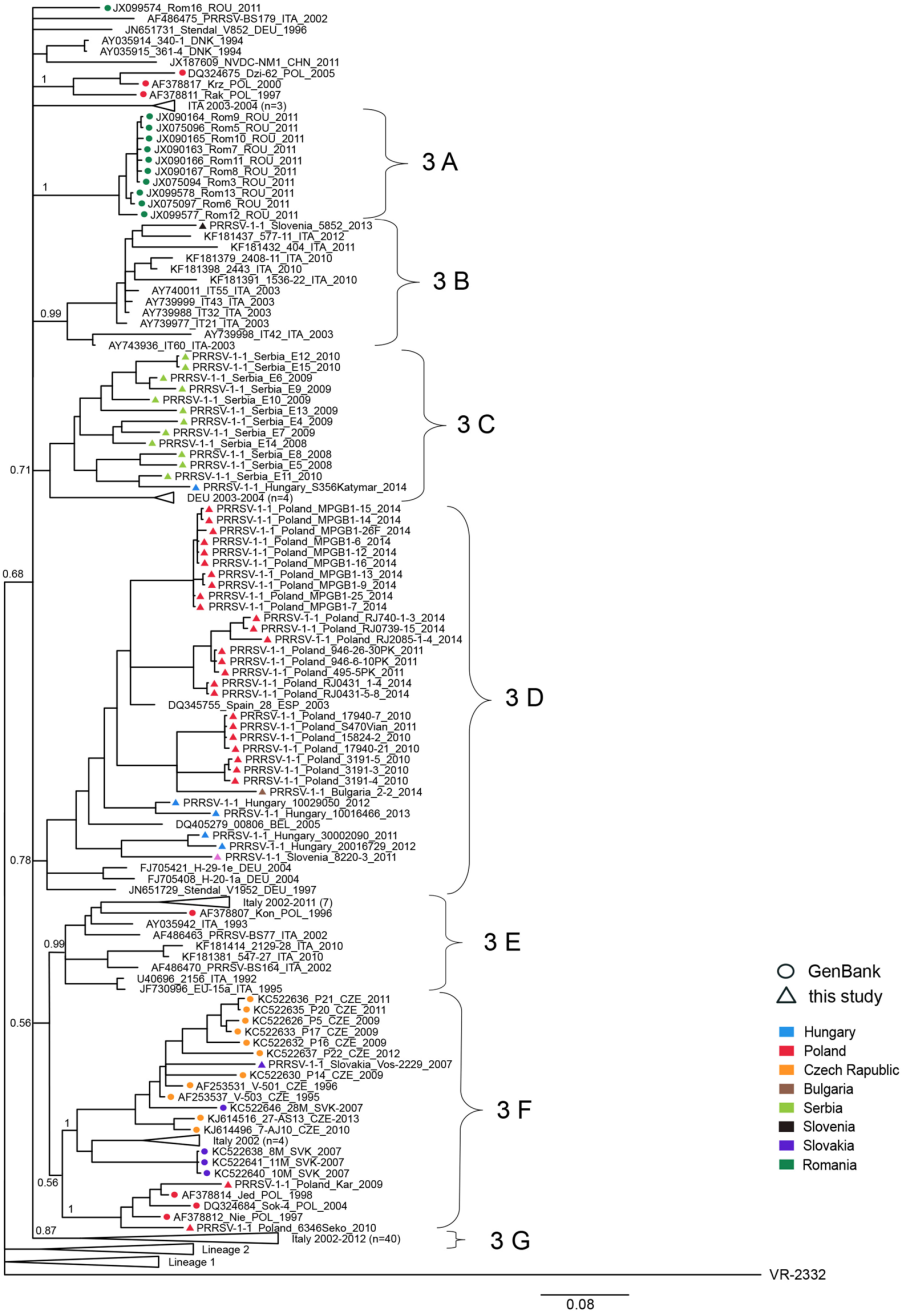
Phylogenetic tree of lineage 3 ORF5 sequences constructed in MrBayes^40^. PRRSV 2 prototype strain VR2332 (AY150564) was used as an outgroup. Some minor clusters were collapsed for clarity. Posterior probability values supporting main nodes were shown. Geographical origin and year of isolation were indicated for each sequence. Braces indicate clades 3 A–3 G.

The oldest sequences from *lineage 3* were identified in Italy in 1992–1993 (Figs 1, 2 and 5). Later two sequences were detected in Denmark in 1994 and two in Germany (1996 and 1997). Since 1995 and 1996 circulation of lineage 3 strains was detected in Czech Republic and Poland, respectively. Apart from that, until 2005 infections with lineage 3 strains were confirmed also in Spain (2003) and Belgium (2005). Further spread to CEE countries was observed after 2007 (Slovakia 2007, Hungary, Slovenia and Romania 2011). All 12 sequences from Serbia (3 from 2008, 6 from 2009 and 3 from 2010) and one sequence from Bulgaria obtained in this study belonged to lineage 3. One lineage 3 sequence from China (2011) indicates also its spread to Asia. What is interesting, no sequences from lineage 3 were detected after 2006 in Western European countries, with the exception of Italy.

Seven well defined clades (3 A–3 G) were identified within lineage 3. Ten Romanian sequences from 2011 formed a separate clade 3 A. The oldest sequences in clades 3 C and 3 D originated from Germany, from 2003 and 1997, respectively. A recent introduction of 3 C strain to Hungary was observed. Also, all 12 sequences from Serbia from 2012 obtained in this study clustered here. In clade 3 D only Western European sequences were identified until 2005. Since 2010 circulation strains from this clade was evident in Poland and Hungary. Additionally, one sequence from Slovenia (2011) and Bulgaria (2014) grouped with this cluster. The oldest strains from clade 3 F were detected in Czech Republic (1995) and continuous circulation of similar strains over the years was confirmed there. Apart from Czech Republic, 3 F strains were detected in Poland (1997–2011) and Slovakia in 2007. Also, four strains were detected in Italy in 2002. Clades 3 B and 3 E are mostly, and clade 3 G exclusively of Italian origin. Apart from Italian sequences, single sequence from Slovenia from 2013 and from Poland 1996 clustered in clade 3 B and 3 E, respectively.

### Movement of live pigs into Central Eastern Europe

Detailed EUROSTAT database research has been conducted regarding live piglet trade tendencies (below 50 kg bodyweight). Altogether 2581954 t in live pig bodyweight was imported into the listed countries within the period of 2004–2013.

The major countries of dispatch to CEE were Germany (311 901 t live pig bodyweight), The Netherlands (265 939 t live pig bodyweight) and Denmark (97 461 t live pig bodyweight). Other countries of origin were only represented by relative low numbers (Table 1).

**Table 1:** Major countries of dispatch regarding live piglet trade (below 50 kg of bodyweight) into CE destinations, according to available EUROSTAT data (2004–2013).

Countries of destination	Major countries of dispatch (100 kg)			
Poland	Germany (2 042 418)	Netherlands (1 167 618)	Denmark (503 724)	Lithuania (135 904)
Hungary	Netherlands (222 803)	Germany (210 554)	Slovakia (30 774)	Lithuania (4 633)
Czech Republic	Denmark (410 886)	Germany (73 322)	Netherlands (71 855)	
Romania	Netherlands (567 417)	Hungary (291 238)	Germany (222 853)	
Slovakia	Netherlands (54 573)	Czech Republic (49 178)	Germany (16 205)	Denmark (15 001)
Slovenia	Austria (86 162)	Netherlands (12 004)	Germany (1 078)	
Bulgaria	Netherlands (23 197)	Germany (6 026)	Romania (1 584)	
Croatia	Germany (546 553)	Netherlands (539 873)	Austria (107 107)	Denmark (45 003)

## Discussion

### Central Eastern European countries differ in terms of dominating genetic lineages of PRRSV 1

The study described in this paper aimed to update the information on the genetic diversity and evolution of PRRSV 1 in Europe, with the special focus on Central Eastern Europe. There is only limited data available on PRRSV diversity from this part of Europe and due to historical and geographical reasons, this region could harbor Eastern European subtypes of PRRSV 1 that are common in the Russian Federation, Ukraine, Belarus, Lithuania and Latvia. The analysis did not reveal any evidence of those Eastern genetic subtypes and only ORF5 sequences belonging to Subtype 1 were detected. However, most of the analyzed sequences originated from Hungary (90), Poland (89) and Czech Republic (23), while only a few were from the remaining countries. Thus, the presence of the Eastern genetic subtypes in CEE cannot be ultimately excluded.

Previous analysis defined 12 genetic clades within Subtype 1 PRRSV^22^. However, genetic recombinations within ORF5^29^ may generate intermediate sequences, blurring the clear-cut division into genetic clades. Here we propose more conservative approach than Shi *et al*.^22^. Three main genetic lineages within Subtype 1 of PRRSV 1 were distinguished and lineages 1 and 3 were further divided into several clades (Figs 1–5). Central Eastern European sequences were found in all three lineages.

Out of 90 sequences from Hungary, 83 (92.2%) were classified in lineage 1 which is also the most common globally since its discovery in 1991^30^. This lineage includes the earliest European PRRSV strains, and at the same time it seems to be well established in Western Europe and increasingly common in Asia. Early evidences of its introduction were also found in those CEE countries (Poland, Czech Republic and Slovakia) where sequences collected before 2000 were available. Some Hungarian sequences, including the oldest ones collected in 2003 formed a distinct clade 1 E, still circulating in Hungary. Hungarian sequences from 2009–2012 identified in clade 1 C and one sequence in clade 1 G (2013) suggest more recent introductions. Two Hungarian sequences (2.2%) from 2012 were detected in lineage 2, most probably as a result of an introduction from Denmark, as large majority of the lineage is formed of Danish sequences and piglets as well as breeding pigs are regularly imported from Denmark into Hungary (data not shown). Additionally, small group of Hungarian sequences (5 sequences, 5.7%) was classified into another major European lineage 3 in clusters 3 C and 3D, where the oldest sequences originated from Germany.

Unfortunately, the earliest Hungarian ORF5 sequences originated from 2003 and 2004 so it can only be speculated that they might have emerged 10 years earlier, when the first evidence of PRRSV in Hungary was reported^25^. This is supported by the results of Medveczky *et al*.^31^, who compared the ORF7 sequence with the corresponding part of 21 other European strains, which confirmed the close relationship between the Hungarian and the Spanish isolates.

In the mid-1990s two genetic lineages, 1 and 3, emerged in Poland. Most of the sequences were classified in lineage 1 (55 out of 89 sequences, 61.8%), and all remaining ones belonged to lineage 3. The earliest Polish sequences from those lineages were from 1994 and 1996, respectively. Fourteen Polish sequences from 1994–2010 formed a distinct cluster 1 D within lineage 1, without any closely similar foreign strains. Relatively recent detection of sequences from clades 1 A, 1B, 1 C and 1 G in Poland (2010–2014) suggests several new introduction events. Around the same time sequences from clade 3 D from lineage 3 were found in Polish herds, together with recent strains from Hungary, Slovenia and Bulgaria. The earliest sequence in this clade – although relatively distant from the Central Eastern European sequences *–* originated from Germany (1997). Five Polish sequences collected between 1997 and 2010 were found in clade 3 F, together with strains from Czech Republic (the oldest in this clade, 1995–2013), Slovakia (2007) and Italy (2002). Interestingly, one early Polish sequence from 1996 was classified in clade 3E, containing exclusively Italian strains collected from 1992 to 2011. Lineage 3 seems to be also common in Romania: 11 out of 17 sequences from 2011 belonged to this lineage.

Also in the Czech Republic lineage 1 and 3 emerged in mid 1990s. Clade 3 F in latter lineage containing 11 Czech sequences from 1995–2013 is particularly interesting as similar strains were only found in the neighboring Poland (5 sequences from 1997–2010), Slovakia (5 sequences from 2007) and in Italy (4 sequences from 2002). Thus, it can be considered a clade of Czech origin, that emerged locally and was already detected as early as 1995 and its spread was very limited. Other early Czech sequences from clade 1 F in lineage 1 were only detected before 1998 and not recently. In the same clade 7 Croatian sequences from 2012 were classified. On the other hand, strains from the same lineage were present in Czech Republic since 2009, suggesting separate introduction. One Czech strain that is located among numerous Danish sequences on the tree was also detected in lineage 2 in 2011. The Danish origin of this strain is also supported by the pig trade data as Demark is the leading country of origin for piglet import into the Czech Republic.

In Serbia only 12 sequences were obtained (3 from 2008, 6 from 2009 and 3 from 2010) and all were classified in the lineage 3, clade 3 C, where also a recent, closely similar sequence from southern Hungary, as well as four, more distant German sequences from 2003–2004 were clustered. The relatively high level of diversity of the Serbian sequences strongly indicates long evolution of the virus before the Serbian pig population was sampled for the purpose of this study. The single sequence in lineage 6 in Hungary was found in a herd located close to the southern border with Serbia indicating regional spread of the strain from Serbia.

In summary in CEE PRRSV strains from two lineages, 1 and 2 emerged in the mid-1990s, and are still circulating. Lineage 1 seems to dominate until present, with 170 sequences (66,9%). In lineages 3 and 2 81 (31.9%) and 3 (1.2%) were classified, respectively. Central Eastern European sequences make 42.4% of the sequences from the lineage 1, 10% of sequences from the lineage 2 and 48.5% of sequences from the lineage 3, where nearly 44% of strains are of Italian origin. Several clades in both lineages 1 and 3, as well as the three sequences from lineage 2 were only detected in CEE in 2009 or later, which may indicate relatively recent introduction (the earlier yet undetected presence however cannot be fully excluded). The differences in distribution of the lineages in Hungary, Poland and Czech Republic indicate different PRRSV history.

### What impacts the genetic diversity of PRRSV observed in CEE?

The main factor that allows PPRSV to spread is the movement of infected animals or semen. It was particularly important in the early years of PRRSV epidemic, and before diagnostic methods were widely available.

Most of the CEE countries were part of a USSR led political and economic block (Eastern Block) from the late 1940s. In 1949, USSR initiated the establishment of the Council for Mutual Economic Assistance (COMECON), an economic organization that was meant to increase collaboration within the Eastern Block. The European members were USSR, Bulgaria, Czechoslovakia, Hungary, Poland, Romania and the German Democratic Republic. This organization existed until 1991. These close economic links, in theory, should have allowed the spread of PRRSV to other COMECON countries as PRRSV emerged and diversified into multiple genetic subtypes in the first years of the 20th century according to a recent ORF5 sequence based molecular clock analysis^32^. However, our findings did not show any evidence that this transmission occurred. The CEE countries were likely protected against PRRSV by the fact that USSR was mainly the importer of agricultural products including pork and live pigs. For example, in the late 1980s 55–60% of the pork export and 90% of the live hog export from Hungary went directly to the USSR^33^. According to the Yearbooks of Foreign Trade Statistics of the Republic of Poland, issued yearly by the Central Statistical Office of Poland, no import of live pigs from USSR to Poland took place during the 1980s and 1990s.

After breaking up of the USSR led COMECON in 1991 and re-orientation of CEE economies towards Western Europe, followed by joining of the EU by some countries, the pig industry showed a dramatic change. The number of sows along with the overall number of pigs raised per year decreased significantly. For example, in Hungary the number of pigs raised annually dropped from 5 million in 2003 to 3 million in 2013. In Poland similar tendencies started in 2008 when in a single year the amount of pigs decreased from almost 19 million (2006) to 14 million (2008) and recently to 10.5 million (2015). The decrease of the number of pigs raised in the Czech Republic showed the most dramatic pattern: it dropped from 3.3 million pigs in the year before joining EU to 1.4 in 2011 and after a slight increase it recently reached around 1.5 million. In Romania the number of pigs raised annually was 6.5 million in 2007 (year of joining EU) which decreased to 5.2 and 4.9 million by 2013 and 2015, respectively (EUROSTAT data).

This drop coincided with the increase in live pig import from the EU. In Hungary for example the import of live pigs in 2005 increased by 250% from 2004 (Hungarian Central Statistical Office). At the same time all of the CEE countries had a significant live swine (piglets below 50 kg) import in this period (see Table 1).

The majority of CEE PRRSV sequences described in this paper are located in lineage 1 where Western European sequences are the oldest, mostly from Spain, the Netherlands, Germany and Denmark. Moreover, we observed the emergence of new PRRSV clades from lineages 1, 2 and 3 in CEE countries in the recent years. Altogether, changes in the structure of pig production in Europe since the fall of the Eastern Block, followed by opening the markets in CEE countries, likely enabled the spread of PRRSV eastward. However, the clade 3 F in lineage 3 is composed mostly of Czech and Slovakian and Polish sequences, with only four examples from Italy. Moreover, the oldest Czech sequences originate from 1995. Czech Republic and Slovakia were parts of Czechoslovakia until the end of 1992, and both countries have a common border with Poland. Thus it is likely that this lineage emerged only locally.

### Impact of modified live vaccines on PRRSV 1 diversity

The impact of the modified live vaccines (MLVs) on the genetic diversity is difficult to assess due to the lack of genetic markers and the fact that the two most commonly used vaccines are based on early strains that were widely spread in Europe before the vaccines were licensed. However, in a recent study the emergence of strains highly similar to a lineage 1 Porcilis PRRS MLV vaccine strain was described in Denmark that coincided with the licensing of this vaccine^21^. These strains had 95.7–99.8% pairwise ORF5 nucleotide identity with the vaccine strain and all of the strains were isolated after Porcilis PRRS had been authorized in Denmark. Thus, the authors suggested that the MLV strain might be circulating among Danish swine herds.

The second PRRSV 1 vaccine strain was also classified in lineage 1 (Amervac, Hipra, Spain). Interestingly two Polish field strains from 1997 share 97% identity in ORF5 with the Amervac strain, which was licensed in Poland only in 2002 (and in 1998 in Spain). Similar strains are being detected in many countries and are often considered to be related to the Amervac vaccine, which may not always be the case.

A recent publication describing the full genome sequence analysis of a virulent Hungarian field isolate (9625/2012) belonging to lineage 1 revealed almost equal similarity to Amervac MLV (96%) and an early wild Spanish isolate Olot1991 (95%)^34^. The authors could not prove unequivocally the MLV or wild type origin of the strain, however the strain was isolated from an outbreak of severe clinical signs, increased mortality rates and marked haemorrhagic post mortem lesions. Since the authors were not aware of any live pig import from Spain to Hungary, this might suggest a possible early introduction and local evolution of a wild type strain from Germany as a plausible ancestral country of lineage 1.

### Tracing the origin of Subtype 1 in Europe

It is clear that PRRSV 1 Subtype 1 diversified before it was detected in early 1990s^19^. This diversification occurred in unknown countries or regions before the individual lineages were distributed internationally. Present data indicates that it has occurred in Western, Central Eastern or Southern, but not in Eastern Europe. Great majority of the Eastern European strains belong to Subtypes 2, 3, 4 or recombinant variants^18^. The present data indicates that the international trade patterns are responsible for the current geographical distribution of the genetic subtypes and lineages of PRRSV 1. The lineage 1 is most common in pig exporting countries like Denmark, Germany and the Netherlands, and is widely distributed in Europe and beyond. Also, lineage 2 strains, previously limited to Denmark, spread to other countries since 2009. On the other hand, clades 3 B and 3 G of lineage 3 are dominating in Italy, from where the export of live pigs is minimal, and those lineages were only occasionally detected outside of Italy. Also, the spread of the clade 3 F was minimal as such strains dominate since at least 1996 in former Czechoslovakia (now Czech Republic and Slovakia), and later were also found in Italy.

The wide distribution of the early lineage 1 strains (sometimes referred to as “Spanish-like”) in Europe, including CEE countries, is interesting. In the 1990s Spain did not export live pigs to other European countries due to their Africal Swine Fever epidemic, but rather imported from Western Europe (Albert Vidal, personal communication). A personal communication by the chief veterinarian of Hungary of that era, Dr. Tibor Bálint, verified that live pigs were officially not imported from Spain into Hungary. Similarly, no live pigs were imported from Spain to Poland in the same period (Yearbooks of Foreign Trade Statistics of the Republic of Poland, 1990–2000). Instead, the import from Western Europe to Spain and CEE countries dominated. Closer analysis of lineage 1 sequences shows that many of the early German sequences are also clustered there (Clade 1 G). Moreover, Germany’s live pig export data from the Statistiches Bundesamt, electronically available from 1996 (kindly provided by Christian Schramm), showed significant live pig export from Germany to Spain, Poland and Hungary among others. Thus, it is justified to suggest that the lineage 1 emerged in Germany and spread to Spain and CEE countries. This conclusion is in conflict with a recent paper published by Nguyen *et al*.^32^, who suggested the opposite direction of PRRSV spread in Europe based on Bayesian analysis of the sequences solely. The authors concluded that the spread and diversification of Subtype 1 strains started from Italy, then the strains were transmitted to Spain and from there to Germany from where they spread across Europe and beyond. However, the authors did not consider the live pigs trade patterns in Europe in the 20^th^ century. We propose that PRRSV 1 Subtype 1 evolved and diversified in multiple locations across Western or Central Eastern Europe, and only strains from major pig exporting countries spread widely.

How the ancestor of PRRSV 1 Subtype 1 reached different countries remains unknown. Since we have more than 20 years of longitudinal sampling covering a wide region, it is tempting to explore the origin of PRRSV 1. The diversity of PRRSV 1 in the former USSR, (presently The Russian Federation, Lithuania, Belarus and Latvia) strongly indicates Eastern European origin of this virus as the greatest genetic diversity of a given organism can always be found at the site of its origin. Interestingly, the identification of Subtype 1 strains in the countries formerly being a part of the USSR is rare and increased only recently (Balka and Stadejek unpublished). This and the fact that USSR and presently Russia, Belarus and Ukraine have not been exporting pigs to Central (including COMECON countries) and Western Europe makes it unlikely that PRRSV transmission occurred through commercial trade.

A possible explanation of PRRSV spread to Western European countries could be that it happened through wild boars. After World War II., but most strikingly from the late 1970s and culminating in 1988 there was an enormous increase in the number of wild boars in the German Democratic Republic^35^. According to letters and reports from this period, the situation was so dramatic that livestock keepers had to patrol their facilities to protect them from interlopers spreading diseases to their animals. The phenomenon was called “die Wildschwein Plage” or wild boar plague. Interestingly, the time and location of the very first PRRSV seropositive samples coincided both geographically (German Democratic Republic) and historically (1987) with this event^23^. Moreover, the very first outbreaks were reported soon after in Germany (1990)^5^.

These events suggest a wild boar related link between Eastern Germany and the hypothetical location of PRRSV origin. Later evidences suggest that only the ancestors of Subtype 1 strains survived the bottleneck effect, and they established the whole PRRSV 1, Subtype 1 population in other parts of Europe and the world. A similar theory was proposed by Peter G. W. Plagemann^36^. A molecular clock of ORF3 suggests the occurrence of a common ancestor of PRRSV 1, that infected domestic pigs about ten years earlier, around 1979^19^. Interestingly, apart from Germany^37^, Lithuania^38^ and the Campania Region of Italy^39^, the wild boar population shows none or only minimal prevalence of PRRSV infections indicating that it rather served as an introducer of PRRSV into the domestic pig population than as a reservoir for continuous infection events.

It could also be speculated that research collaboration of USSR animal husbandry institutes or faculties from COMECON or Western European counterparts in 1960s or 1970s allowed for a very limited transmission of PRRSV that later evolved into Subtype 1, and spread in Europe, while in USSR other subtypes evolved and spread in the whole area of the large state from borders of Poland, Slovakia, Hungary and Romania in the west to the Far East. Together with the genetic evidences, those plausible scenarios may indicate that Germany was a PRRSV hub in Western and Central Europe, apart from Italy.

Summarizing the data obtained in this study, some new features were identified regarding the evolution and spread of PRRSV in Central Eastern European countries. However, it has to be stressed that the overall phylogenetic diversity in CEE and also in Europe is far from being complete as there are lots of missing pieces in the picture, mostly due to the low number of early (except Poland), and recent PRRSV sequences (except Poland and Hungary). We believe that our results can be important for researchers and stakeholders in Europe to use it as a tool for further sequence classifications and epidemiological analyses. It can also be useful in comprehensive PRRS control and elimination programs. As a part of the National PRRS Eradication Program launched in January 2014, Hungary started a project to get a complete ORF5 phylogenetic mapping of every known PRRS positive pig herd in the country.

The new classification system along with the provided set of Subtype 1 sequences might help to harmonize the phylogenetic nomenclature of PRRSV 1. Moreover our data might help to evaluate the origin of the strains and to trace the origin of the virus in new outbreaks especially in the countries of CEE where minimal information was available so far. Such an epidemiological investigation can be utilized to find critical points in the biosecurity of the given farms and implement changes in the others to prevent such events.

To the authors’ knowledge this is the first publication regarding PRRSV 1 phylogeny where actual live pig trade data were analyzed in parallel. Without such data, phylogenetic analysis even with the most advanced bioinformatics approaches alone can be misleading. In a recent paper based on Bayesian analysis alone Nguyen *et al*.^32^ showed transmission events that – according to our data – most likely did not happen and they referred to Spain as a viral intermediate distribution source. In contrast, our findings suggest the same role for Germany which is highly supported by the actual trade patterns. It has to be highlighted though that our knowledge on genetic diversity of PRRSV in Europe, and especially in Central Eastern Europe, is very limited. So, the history of PRRSV evolution in this region remains to be elucidated. The tool can only be improved and developed based on the analysis of continuously updated systematic sequence collections (like in the USA), thus the authors encourage everybody involved in PRRSV sequencing in Europe to deposit as many sequences as possible to the GenBank.

## Materials and Methods

### Sample collection and DNA sequencing

Various samples of serum, aborted fetuses or lung tissues from pigs were submitted to the authors’ laboratories as a part of routine diagnostic procedures and/or monitoring programs and tested for the presence of PRRSV with locally used various diagnostic PCR methods. No intentional animal handlings and/or samplings have been carried out for the purposes of this study: the samples we used had been archived previously according to the different laboratories’ protocols. Every diagnostic sample originating from live animal has been obtained in accordance with the different countries’ ethical and animal welfare regulations.

Complete ORF5 from the selected samples was amplified and sequenced with the Sanger method. For a more detailed analysis previously published ORF5 sequences from Hungary, Poland, Czech Republic, Slovakia and Slovenia were downloaded from the GenBank. Altogether 254 sequences from Central Eastern Europe were obtained: 54 from Hungary (plus 36 previously published), 68 from Poland (plus 21 previously published), 7 from Croatia, 23 from Czech Republic, 17 previously published from Romania, 2 from Slovakia (plus 6 previously published), 7 from Slovenia, 12 from Serbia and 1 from Bulgaria (GenBank accession numbers: MF600473–MF600623).

### Sequence analysis

The ORF5 sequences from Central Eastern Europe were analyzed together with the ORF5 of PRRSV 1 Subtype 1 representing the full range of genetic diversity of this Subtype. Altogether 607 sequences were analyzed in the study (Supplementary Table). Sequence alignment was performed based on ClustalW (Geneious 10.1.3, Biomatters) and phylogenetic analysis using a Bayesian Markov chain Monte Carlo method in MrBayes v3.2^40^. Detailed parameter settings and the principles of lineage definitions were identical to those described previously^41^. A general time-reversible nucleotide substitution model with 4 categories of gamma-distributed rate heterogeneity and a proportion of invariant sites GTR (GTR + Г4 + I) was used. The posterior distribution of trees and model parameters were summarized from Markov chain Monte Carlo sampling over 1.1 million generations, during which trees were sampled every 250 generations. The initial 10% of samples were discarded as burn-in.

### Movement of live pigs in Europe

To investigate the influence of live pig trading tendencies on PRRSV diversity over the years we conducted a detailed search in the available database of the European Commission’s Directorate-General responsible for statistics (EUROSTAT).

We have focused on the reporter countries, where the live swine consignments were dispatched to. The reporter country is the Member State of the European Union which reports the trade data to EUROSTAT. Partner countries are the countries from where the live swine consignments were dispatched to the reporter countries. During our queries we focused only on the trade from EU-28.

Briefly the search was conducted under the theme’International trade’, using the harmonized system code 0103 (tariff nomenclature code for live swine). The customized queries contained annual time series from 2004 to 2013. The quantities are presented in 100 kg units. Data from 2014 was not available at the time of the query, thus, the selected time period was from 2004 to 2013.

## Electronic supplementary material

Supplementary Table
