# Supplementary material for: Genetic diversity of PRRSV 1 in Central Eastern Europe in 1994–2014: origin and evolution of the virus in the region
Source: Sci Rep. 2018 May 17;8:7811. doi: 10.1038/s41598-018-26036-w (PMC5958080; doi:10.1038/s41598-018-26036-w)
Supplement: Supplementary file 1 — Supplementary Table [file 41598_2018_26036_MOESM1_ESM.pdf]

Genetic diversity of PRRSV 1 in Central Eastern Europe in 1994-2014: origin and evolution of the virus in the region

Gyula Balka[1*], Katarzyna Podgórska[2], Manreet Brar[3], Ádám Bálint[4], Daniel Cadar[5], Vladimir Celer[6], Lilla Dénes[1], Zuzana Dirbakova[7], Anna Jedryczko[8], Lázár Márton[9], Dinko Novosel[10], Tamas Petrovic[11], Ivo Sirakov[12], Dóra Szalay[4], Ivan Toplak[13], Frederick Chi-Ching Leung[3], Tomasz Stadejek[14]

[1] Department of Pathology, University of Veterinary Medicine, István Str. 2, H-1076 Budapest, Hungary

[2] National Veterinary Research Institute, Partyzantów 57, 24-100 Pulawy, Poland

[3] The University of Hong Kong, 5N-12, Kadoorie Biological Science Building, Hong Kong, China

[4] National Food Chain Safety Office Veterinary Diagnostic Directorate, Tábornok Str. 2, H-1149 Budapest, Hungary

[5]Arbovirology, Bernhard Nocht Institute for Tropical Medicine, WHO Collaborating Centre for Arbovirus and Haemorrhagic Fever Reference and Research National Reference Centre for Tropical Infectious Diseases, Bernhard Nocht Strasse 74, 20359 Hamburg, Germany

[6] Faculty of Veterinary Medicine, Institute of Infectious Diseases and Microbiology, University of Veterinary and Pharmaceutical Sciences Brno, 612 42 Brno, Czech Republic

[7] Veterinary Institute Zvolen, Pod Drahami 918, 960 86 Zvolen, Slovak Republik

[8] Veterinary Diagnostic Laboratory, Ostródzka, 11-036 Gietrzwałd, Poland

[9] National Food Chain Safety Office Animal Health and Animal Welfare Directorate, Keleti Károly Str. 24, H-1024 Budapest, Hungary

[10] University of Zagreb, Faculty of Agriculture, Department of Animal Science, Svetošimunska cesta 25, 10000 Zagreb, Croatia

[11] Scientific Veterinary Institute „Novi Sad“ Rumenački put 20, 21000 Novi Sad, Serbia

[12] Department of Medical Microbiology, Medical University of Sofia, Zdrave Str. 2, 1431 Sofia, Bulgaria

[13] University of Ljubljana Veterinary Faculty National Veterinary Institute Gerbičeva 60 1000 Ljubljana, Slovenia

[14] Department of Pathology and Veterinary Diagnostics, Faculty of Veterinary Medicine, Warsaw University of Life Sciences – SGGW, ul. Nowoursynowska 159c, 02-776 Warsaw, Poland

*Correspondence should be addressed to Gy. B. (balka.gyula@univet.hu)

Supplementary Table List of sequences used to generate the phylogenetic tree.

Country	Sequence ID	Year of isolation *if not known - year of submission	GenBank Nos of sequences obtained in this study	Lineage	Clade
AUT	AY615791_LU536	*2004		1	
AUT	AY615792_SE551	*2004		1	
AUT	AY875856_2810_na	*2005		1	
AUT	AY875861_1935	*2005		1	
AUT	AY875862_2906_2	*2005		1	
AUT	AY875857_2783	*2005		1	
AUT	AY875853_1695	*2005		1	
AUT	AY875860_2103	*2005		1	
AUT	KC522617_LA2	*2005		1	
AUT	AY875859_2231	*2005		1	
AUT	KC522619_LA6	2008		1	
AUT	KC522621_LA9	2008		1	
AUT	KC522618_LA3	2008		1	
AUT	KC522616_LA1	2008		1	
AUT	KC522620_LA8	2008		1	
BEL	U40700_AV30	*1995		1	
BEL	JF730992_EU-9a_(02V48)	2002		1	
BEL	DQ405282_2005_86AS	2005		1	
BEL	DQ405286_2005_85-1AS	2005		1	
BEL	DQ405284_1413	*2006		1	
BEL	DQ405279_2005_00806	2005		3	3D
BUL	PRRSV-1-1_Bulgaria_2-2	2014	MF600473	3	3D
CHN	GQ461593_SHE	*2009		1	
CHN	KF001144_GZ11-G1	2011		1	
CHN	AY633973_B13	1999		1	1F
CHN	JX187609_NVDC-NM1	2011		3	
CHN	GU047340_NMEU09-2	2009		2	
CHN	GU047341_NMEU09-3	2009		2	
CHN	GU047343_NMEU09-5	2009		2	
CRO	PRRSV-1-1_Croatia_4	2012	MF600477	1	1F
CRO	PRRSV-1-1_Croatia_8	2012	MF600479	1	1F
CRO	PRRSV-1-1_Croatia_1	2012	MF600474	1	1F
CRO	PRRSV-1-1_Croatia_3	2012	MF600476	1	1F
CRO	PRRSV-1-1_Croatia_5	2012	MF600478	1	1F
CRO	PRRSV-1-1_Croatia_2	2012	MF600475	1	1F
CRO	PRRSV-1-1_Croatia_H3	2014	MF600480	1	1F

CZE	KJ614495_6-AB09	2009		1	
CZE	KC522625_P4	2009		1	
CZE	KC522628_P9	2009		1	
CZE	KJ614506_17-D12	2012		1	
CZE	KJ614510_21-A12	2012		1	
CZE	KJ614514_25-A12	2012		1	
CZE	KJ614519_30-A13	2013		1	
CZE	KJ614523_34-A14	2014		1	
CZE	AF253535_V-546	1995		1	1F
CZE	AF253532_V-502	1996		1	1F
CZE	AF253534_V-548	1998		1	1F
CZE	AF253537_V-503	1995		3	3F
CZE	AF253531_V-501	1996		3	3F
CZE	KC522626_P5	2009		3	3F
CZE	KC522633_P17	2009		3	3F
CZE	KC522632_P16	2009		3	3F
CZE	KC522630_P14	2009		3	3F
CZE	KJ614496_7-AJ10	2010		3	3F
CZE	KC522636_P21	2011		3	3F
CZE	KC522635_P20	2011		3	3F
CZE	KC522637_P22	2012		3	3F
CZE	KJ614516_27-AS13	2013		3	3F
CZE	KJ614499_10-P11	2011		2	
DEU	JF730985_EU-2a	1992		1	
DEU	AY035923_2.46_1993	1993		1	
DEU	AY035921_2.25_1993	1993		1	
DEU	JN651728_Stendal_V953	1996		1	
DEU	JN651739_BH_95_10-13	2000		1	
DEU	AF378798_AS1	2001		1	
DEU	FJ705430_H-47-1a	2004		1	
DEU	FJ705380_H-02-1b	2004		1	
DEU	FJ705423_H-30-1b	2004		1	
DEU	FJ705381_H-02-1e	2004		1	
DEU	FJ705412_H-25-1a	2005		1	
DEU	FJ705417_H-26-2d	2006		1	
DEU	FJ705416_H-26-2c	2006		1	
DEU	JN651730_Cobbelsdorf	1991		1	1F
DEU	AF378797_Arnsberg_NA	*2001		1	1F
DEU	AY035925_2.96	1993		1	1G
DEU	JN651732_Stendal_V1904	1996		1	1G
DEU	JN651737_BH_95_10-08_EU	2002		1	1G
DEU	FJ705377_H-01-2a	2003		1	1G
DEU	FJ705383_H-03-1a	2004		1	1G
DEU	FJ705384_H-03-1b	2004		1	1G
DEU	FJ705395_H-06-1d	2004		1	1G

DEU	FJ705397_H-06-1g	2004		1	1G
DEU	FJ705411_H-23-1a	2004		1	1G
DEU	FJ705401_H-13-1a	2004		1	1G
DEU	FJ705429_H-46-1a	2004		1	1G
DEU	JN651738_BH_95_10-12	2006		1	1G
DEU	JN651731_Stendal_V852	1996		3	
DEU	FJ705373_H-01-1d	2003		3	3C
DEU	FJ705375_H-01-1f	2003		3	3C
DEU	FJ705376_H-01-1g	2003		3	3C
DEU	FJ705391_H-05-1a	2004		3	3C
DEU	JN651729_Stendal_V1952_97	1997		3	3D
DEU	FJ705421_H-29-1e	2004		3	3D
DEU	FJ705408_H-20-1a	2004		3	3D
DNK	AY035909_228	1993		1	
DNK	AY035917_5767-6	1995		1	
DNK	KC862531_DK-2003-8-2	2003		1	
DNK	KC862526_DK-2008-10-5-3	2008		1	
DNK	KC862568_DK-2010-10-10-3	2010		1	
DNK	KC862564_2010	2010		1	
DNK	KC862562_DK-2010-30-11-11	2010		1	
DNK	KC862569_DK-2011-05-23-9	2011		1	
DNK	KC862504_DK-2011-05-11-3	2011		1	
DNK	KC862517_DK-2011-09-02-2	2011		1	
DNK	KC862507_DK-2011-05-11-20	2011		1	
DNK	KC862520_DK-2012-10-9-3	2012		1	
DNK	KC862519_DK-2012-30-8-8	2012		1	
DNK	KC862518_DK-2012-01-05-11	2012		1	
DNK	KC862525_DK-2013-30-3-6	2013		1	
DNK	KC862524_DK-2013-10-2-1	2013		1	
DNK	AY035914_340-1	1994		3	
DNK	AY035915_361-4	1994		3	
DNK	AY035913_32-10_92	1992		2	
DNK	AY035916_48-1_92_	1992		2	
DNK	KC862566_DK-1992-PRRS-111_92	1992		2	
DNK	AY035944	1992		2	
DNK	AJ223078_Danish_DK111-92	1992		2	
DNK	AY035906_18794	1993		2	
DNK	AY035903_12770_95	1995		2	
DNK	AY035902_12654	1995		2	
DNK	AY035904_12985	1996		2	
DNK	AY035905_14474B	1996		2	
DNK	AY035908_21191	1997		2	
DNK	AY035910_24554_97	1997		2	
DNK	AY035907_20567	1997		2	
DNK	AY035912_28639_98	1998		2	

DNK	KC862530_DK-2003-6-2	2003		2	
DNK	KC862571_DK-2003-6-5	2003		2	
DNK	KC862572_DK-2003-7-2	2003		2	
DNK	KC862528_DK-2007-10-1-3	2007		2	
DNK	KF662722_DK-2007-10-1-1	2007		2	
DNK	KC862529_DK-2007-80-2-1	2007		2	
DNK	KC862508_DK-2011-05-23-2	2011		2	
DNK	KC862515_DK-2011-30-9-13	2011		2	
DNK	KC862521_DK-2012-10-01-27	2012		2	
DNK	KC862522_DK-2012-08-21-32	2012		2	
ESP	DQ345728_Spain_1	1991		1	
ESP	U40687_571091	1991		1	
ESP	U40702_5710	1991		1	
ESP	KC862570_ESP-1991-Olot91	1991		1	
ESP	U40691_5999	1991		1	
ESP	U40690_4606	1991		1	
ESP	AY035936_65_2_91	1991		1	
ESP	DQ345735_Spain_8	1992		1	
ESP	DQ345733_Spain_6	1992		1	
ESP	DQ345734_Spain_7	1992		1	
ESP	DQ345732_5	1992		1	
ESP	U40689_3211	1992		1	
ESP	DQ345738_Spain_11	1994		1	
ESP	GQ451664_CReSA-3012	1997		1	
ESP	DQ345740_Spain_13	2000		1	
ESP	DQ345741_Spain_14	2000		1	
ESP	DQ345745_Spain_18	2000		1	
ESP	DQ345747_Spain_20	2002		1	
ESP	DQ345752_Spain_25	2002		1	
ESP	DQ345748_Spain_21	2002		1	
ESP	GQ451663_CReSA-2988	2003		1	
ESP	DQ345753_Spain_26	2003		1	
ESP	GQ451672_CReSA-3256	2005		1	
ESP	GQ451670_CReSA-2982	2005		1	
ESP	GQ451662_CReSA-2755	2005		1	
ESP	KF666907_CReSA-47	2007		1	
ESP	KF666904_CReSA-44	2007		1	
ESP	KF666908_CReSA-48	2008		1	
ESP	KF666913_CReSA-53	2008		1	
ESP	KF666912_CReSA-52	2008		1	
ESP	KF666914_CReSA-54	2008		1	
ESP	GQ451673_V3_VACC	*2009		1	
ESP	KF666919_CReSA-59	2009		1	
ESP	KF666918_CReSA-58	2009		1	
ESP	KF666916_CReSA-56	2009		1	

ESP	KF666917_CReSA-57	2009		1	
ESP	KF666936_CReSA-76	2010		1	
ESP	KF666942_CReSA-82	2010		1	
ESP	KF666949_CReSA-89	2010		1	
ESP	KF666944_CReSA-84	2010		1	
ESP	KF666928_CReSA-68	2010		1	
ESP	KF666933_CReSA-73	2010		1	
ESP	D5_KF666930_CReSA-70	2010		1	
ESP	KF666937_CReSA-77	2010		1	
ESP	KF666921_CReSA-61	2010		1	
ESP	KF666940_CReSA-80	2010		1	
ESP	KF666948._CReSA-88	2010		1	
ESP	DQ345737_Spain_10	2993		1	
ESP	AY035937_L51_2_92	1992		1	1G
ESP	U40688_2228	1992		1	1G
ESP	DQ345739_Spain_12	1995		1	1G
ESP	U40694_P035	1995		1	1G
ESP	DQ345742_Spain_15	2000		1	1G
ESP	DQ345751_Spain_24	2002		1	1G
ESP	DQ345750_Spain_23	2002		1	1G
ESP	DQ345754_Spain_27	2003		1	1G
ESP	GQ451665_CReSA-2653	2005		1	1G
ESP	GQ451668_CReSA-2987	2005		1	1G
ESP	KF666910_CReSA-50	2007		1	1G
ESP	KF666909_CReSA-49	2007		1	1G
ESP	KF666906_CReSA-46	2008		1	1G
ESP	KF666920_CReSA-60	2009		1	1G
ESP	KF666915_CReSA-55	2009		1	1G
ESP	KF666946_CReSA-86	2010		1	1G
ESP	KF666934_CReSA-74	2010		1	1G
ESP	KF666935_CReSA-75	2010		1	1G
ESP	KF666945_CReSA-85	2010		1	1G
ESP	KF666939_CReSA-79	2010		1	1G
ESP	KF666929_CReSA-69	2010		1	1G
ESP	DQ345755_Spain_28	2003		3	3D
FRA	AY035918_SDRPII7A	1991		1	
FRA	AY035919_SDRPIV4A	1992		1	
FRA	GQ451666_CReSA-2744	1993		1	
FRA	AY035920_SDRPV4A	1993		1	
FRA	U40697_5A	1996		1	
FRA	U40699_8D	1996		1	
FRA	U40698_6A	1996		1	
GBR	JN862378_NY1	1991		1	
GBR	JN862376_H4	1991		1	
GBR	JN862377_H5	1991		1	

GBR	JN862380_NY3	1991		1	
GBR	AY035938_H2-D768	1991		1	
GBR	JN862379_NY2	1991		1	
GBR	JN862382_Le1	1992		1	
GBR	JN862384_Su1	1992		1	
GBR	AY035939_L1-D767	1992		1	
GBR	JN862383_No1	1992		1	
GBR	AY035940_NY3-D769	1992		1	
GBR	JN862381_Ha1	1992		1	
GBR	JN862385_Be1	1993		1	
GBR	JN862386_NY4	1994		1	
GBR	JN862388_2088-03	2003		1	
GBR	JN862395_801-04	2004		1	
GBR	JN862399_52-05	2004		1	
GBR	JN862394_797-04	2004		1	
GBR	JN862389_57-04	2004		1	
GBR	JN862393_796-04	2004		1	
GBR	JN862419_471-05	2005		1	
GBR	JN862414_437-05	2005		1	
GBR	JN862436_1395-05	2005		1	
GBR	JN862408_231-05	2005		1	
GBR	JN862424_678-05	2005		1	
GBR	JN862401_172-05	2005		1	
GBR	JN862434_1391-05	2005		1	
GBR	JN862404_195-05	2005		1	
GBR	JN862407_219-05	2005		1	
GBR	JN862402_173-05	2005		1	
GBR	JN862455_267-06	2006		1	
GBR	JN862459_497-06	2006		1	
GBR	JN862452_244-06	2006		1	
GBR	JN862445_230-06	2006		1	
GBR	JN862460_727-06	2006		1	
GBR	JN862486_684-07	2007		1	
GBR	JN862470_39-07	2007		1	
GBR	JN862487_804-07	2007		1	
GBR	JN862490_998-07	2007		1	
GBR	JN862472_179-07	2007		1	
GBR	JN862484_544-07	2007		1	
GBR	JN862475_224-07	2007		1	
GBR	JN862492_15-08	2008		1	
GBR	JN862498_650-08	2008		1	
GBR	JN862501_708-08	2008		1	
GBR	JN862493_16-08	2008		1	
HKG	KF287129_HK3	2003		1	1B
HKG	KF287130_HK5	2004		1	1B

HKG	KF287131_HK10	2004		1	1B
HKG	KF287128_HK8	2004		1	1B
HUN	DQ366657_HU19	2004		1	
HUN	DQ366654_HU16	2004		1	
HUN	DQ366651_HU13	2004		1	
HUN	EF406347_HU32	2004		1	
HUN	DQ366652_HU14	2004		1	
HUN	EF406350_HU35	2005		1	
HUN	EF406351_HU36	2005		1	
HUN	EF406352_HU37	2005		1	
HUN	DQ384986_HUN22	2005		1	
HUN	EF406349_HU34	2005		1	
HUN	DQ366647_HU09	2005		1	
HUN	DQ384984_HUN18	2005		1	
HUN	DQ384989_HUN26	2005		1	
HUN	DQ384988_HUN25	2005		1	
HUN	DQ366648_HU10	2005		1	
HUN	DQ366649_HU11	2005		1	
HUN	PRRSV-1-1_Hungary_20009701	2008	MF600506	1	
HUN	JF730995_EU-14a	2008		1	
HUN	PRRSV-1-1_Hungary_10017358	2009	MF600493	1	
HUN	PRRSV-1-1_Hungary_10017729	2009	MF600494	1	
HUN	PRRSV-1-1_Hungary_10008520	2010	MF600484	1	
HUN	PRRSV-1-1_Hungary_20018803	2011	MF600518	1	
HUN	PRRSV-1-1_Hungary_20017257	2011	MF600517	1	
HUN	PRRSV-1-1_Hungary_10016969	2011	MF600492	1	
HUN	PRRSV-1-1_Hungary_300103340	2012	MF600531	1	
HUN	PRRSV-1-1_Hungary_20003411	2012	MF600503	1	
HUN	PRRSV-1-1_Hungary_10016168	2012	MF600490	1	
HUN	PRRSV-1-1_Hungary_10014503	2012	MF600489	1	
HUN	PRRSV-1-1_Hungary_10013966	2012	MF600488	1	
HUN	PRRSV-1-1_Hungary_20009880	2012	MF600508	1	
HUN	PRRSV-1-1_Hungary_20009784	2012	MF600507	1	
HUN	PRRSV-1-1_Hungary_200001625	2013	MF600527	1	
HUN	PRRSV-1-1_Hungary_20014565	2013	MF600512	1	
HUN	PRRSV-1-1_Hungary_20013657	2013	MF600511	1	
HUN	PRRSV-1-1_Hungary_100003078	2013	MF600524	1	
HUN	PRRSV-1-1_Hungary_100001309	2013	MF600523	1	
HUN	PRRSV-1-1_Hungary_20000912	2013	MF600502	1	
HUN	PRRSV-1-1_Hungary_200003274	2013	MF600528	1	
HUN	PRRSV-1-1_Hungary_200004091	2013	MF600529	1	
HUN	PRRSV-1-1_Hungary_20000780	2013	MF600501	1	
HUN	PRRSV-1-1_Hungary_202	2014	MF600481	1	
HUN	PRRSV-1-1_Hungary_S048-14-Szil	2014	MF600533	1	
HUN	DQ366656_HU18	2004		1	1A

HUN	DQ366646_HU08	2005		1	1A
HUN	PRRSV-1-1_Hungary_10030640	2011	MF600499	1	1A
HUN	PRRSV-1-1_Hungary_10008705	2012	MF600485	1	1A
HUN	PRRSV-1-1_Hungary_10030775	2012	MF600500	1	1A
HUN	PRRSV-1-1_Hungary_100003712	2013	MF600525	1	1A
HUN	PRRSV-1-1_Hungary_10018120	2013	MF600495	1	1A
HUN	PRRSV-1-1_Hungary_10006324	2009	MF600483	1	1C
HUN	PRRSV-1-1_Hungary_10027406	2012	MF600497	1	1C
HUN	EF406340_HU25	2003		1	1E
HUN	DQ366640_HU02	2003		1	1E
HUN	DQ366639_HU01	2003		1	1E
HUN	DQ366642_HU04	2003		1	1E
HUN	DQ366643_HU05	2004		1	1E
HUN	EF406342_HU27	2004		1	1E
HUN	EF406343_HU28	2004		1	1E
HUN	EF406344_HU29	2004		1	1E
HUN	EF406345_HU30	2004		1	1E
HUN	DQ366644_HU06	2004		1	1E
HUN	EF406346_HU31	2004		1	1E
HUN	DQ366653_HU15	2004		1	1E
HUN	DQ366641_HU03	2004		1	1E
HUN	EF406341_HU26	2004		1	1E
HUN	DQ366645_HU07	2005		1	1E
HUN	DQ366658_HU20	2005		1	1E
HUN	DQ366655_HU17	2005		1	1E
HUN	PRRSV-1-1_Hungary_20026017	2010	MF600521	1	1E
HUN	PRRSV-1-1_Hungary_20015223	2011	MF600514	1	1E
HUN	PRRSV-1-1_Hungary_660	2011	MF600482	1	1E
HUN	PRRSV-1-1_Hungary_10024451	2011	MF600496	1	1E
HUN	PRRSV-1-1_Hungary_20012231	2012	MF600509	1	1E
HUN	PRRSV-1-1_Hungary_20015641	2012	MF600515	1	1E
HUN	PRRSV-1-1_Hungary_20018929	2012	MF600519	1	1E
HUN	PRRSV-1-1_Hungary_20009470	2012	MF600505	1	1E
HUN	PRRSV-1-1_Hungary_20008442	2012	MF600504	1	1E
HUN	PRRSV-1-1_Hungary_20014781	2012	MF600513	1	
HUN	PRRSV-1-1_Hungary_10009143	2012	MF600486	1	
HUN	PRRSV-1-1_Hungary_10009625	2012	MF600487	1	
HUN	PRRSV-1-1_Hungary_200004505	2013	MF600530	1	1E
HUN	PRRSV-1-1_Hungary_Pankota	2014	MF600532	1	1E
HUN	PRRSV-1-1_Hungary_100004659	2013	MF600526	1	1G
HUN	PRRSV-1-1_Hungary_S356Katymar	2014	MF600534	3	3C
HUN	PRRSV-1-1_Hungary_30002090	2011	MF600522	3	3D
HUN	PRRSV-1-1_Hungary_10029050	2012	MF600498	3	3D
HUN	PRRSV-1-1_Hungary_20016729	2012	MF600516	3	3D
HUN	PRRSV-1-1_Hungary_10016466	2013	MF600491	3	3D

HUN	PRRSV-1-1_Hungary_20012780	2012	MF600510	2	
HUN	PRRSV-1-1_Hungary_20020250	2012	MF600520	2	
ITA	AY035927_1751_93	1993		1	
ITA	AY035926_1_93	1993		1	
ITA	AF486464_PRRSV-BS82	*2002		1	
ITA	KF181421_1040-39	2010		1	
ITA	KF181395_390-45	2010		1	
ITA	AF486475_PRRSV-BS179	*2002		3	
ITA	AY739961_IT5	2003		3	
ITA	AY739960_IT4	2004		3	
ITA	AY739973_IT17	2004		3	
ITA	AY740011_IT55	2003		3	3B
ITA	AY739999_IT43	2003		3	3B
ITA	AY739988_IT32	2003		3	3B
ITA	AY739977_IT21	2003		3	3B
ITA	AY739998_IT42	2003		3	3B
ITA	AY743936_IT60	2003		3	3B
ITA	KF181379_2408-11	2010		3	3B
ITA	KF181398_2443	2010		3	3B
ITA	KF181391_1536-22	2010		3	3B
ITA	KF181432_404	2011		3	3B
ITA	KF181437_577-11	2012		3	3B
ITA	U40696_2156	1992		3	3E
ITA	AY035942	1993		3	3E
ITA	JF730996_EU-15a	1995		3	3E
ITA	AY740008_IT52	2002		3	3E
ITA	AY740007_IT51	2002		3	3E
ITA	AF486463_PRRSV-BS77	*2002		3	3E
ITA	AF486470_PRRSV-BS164	*2002		3	3E
ITA	AY740005_IT49	2003		3	3E
ITA	AY740004_IT48	2003		3	3E
ITA	AY740006_IT50	2003		3	3E
ITA	AY739963_IT7	2004		3	3E
ITA	KF181414_2129-28	2010		3	3E
ITA	KF181381_547-27	2010		3	3E
ITA	KF181400_716-28	2011		3	3E
ITA	AF486477_PRRSV-BS186	*2002		3	3F
ITA	AF486476_PRRSV-BS180	*2002		3	3F
ITA	AF486466_PRRSV-BS85	*2002		3	3F
ITA	AF486455_PRRSV-BS37	*2002		3	3F
ITA	AY739982.1	2002		3	3G
ITA	AY739983.1	2002		3	3G
ITA	AY739976.1	2003		3	3G
ITA	AY739985.1	2003		3	3G
ITA	AY739965.1	2003		3	3G

ITA	AY740012.1	2003		3	3G
ITA	AY739981.1	2003		3	3G
ITA	AY739980.1	2003		3	3G
ITA	AY739972.1	2003		3	3G
ITA	AY739969.1	2003		3	3G
ITA	AY739979.1	2003		3	3G
ITA	AY743937.1	2003		3	3G
ITA	AY739964.1	2004		3	3G
ITA	AY739984.1	2004		3	3G
ITA	KF181403.1	2010		3	3G
ITA	KF181409.1	2010		3	3G
ITA	KF181410.1	2010		3	3G
ITA	KF181408.1	2010		3	3G
ITA	KF181387_162-32	2010		3	3G
ITA	KF181425.1	2010		3	3G
ITA	KF181392.1	2010		3	3G
ITA	KF181382.1	2010		3	3G
ITA	KF181399.1	2010		3	3G
ITA	KF181419.1	2010		3	3G
ITA	KF181396.1	2010		3	3G
ITA	KF181443.1	2011		3	3G
ITA	KF181389.1	2011		3	3G
ITA	KF181397.1	2011		3	3G
ITA	KF181436.1	2011		3	3G
ITA	KF181413.1	2011		3	3G
ITA	KF181402.1	2011		3	3G
ITA	KF181423.1	2011		3	3G
ITA	KF181430.1	2011		3	3G
ITA	KF181440.1	2011		3	3G
ITA	KF181384.1	2011		3	3G
ITA	KF181435.1	2012		3	3G
ITA	KF181427.1	2012		3	3G
ITA	KF181426.1	2012		3	3G
ITA	KF181439.1	2012		3	3G
ITA	KF181431.1	2012		3	3G
KOR	JQ656061_K08-0502	2008		1	
KOR	JQ656037_K08-0343	2008		1	
LTU	KC714028_Kre	2013		1	
LVA	KC714036_Ulb-64	2009		1	
NLD	U40695_NL3	1992		1	
NLD	KJ127878_MLV-DV	1999		1	
NLD	M96262_Lelystad	1991		1	1F
NLD	L04493_Boxmeer_10	1992		1	1F
POL	AF378813_Sma	1997		1	
POL	AF378804_Lek	1997		1	

POL	AF378815_Gra	2000		1	
POL	PRRSV-1-1_Poland_Gol-11	2009	MF600566	1	
POL	PRRSV-1-1_Poland_1646-78	2010	MF600546	1	
POL	PRRSV-1-1_Poland_1267-3	2010	MF600545	1	
POL	PRRSV-1-1_Poland_5458-1	2010	MF600552	1	
POL	PRRSV-1-1_Poland_3407-41	2010	MF600551	1	
POL	PRRSV-1-1_Poland_1098-10-1	2011	MF600538	1	
POL	PRRSV-1-1_Poland_1098-10-2	2011	MF600539	1	
POL	PRRSV-1-1_Poland_01104-14	2011	MF600541	1	
POL	PRRSV-1-1_Poland_1098-16	2011	MF600540	1	
POL	PRRSV-1-1_Poland_01104-19	2011	MF600543	1	
POL	PRRSV-1-1_Poland_01104-16	2011	MF600542	1	
POL	PRRSV-1-1_Poland_S358Vian	2011	MF600600	1	
POL	PRRSV-1-1_Poland_S473Vian	2011	MF600602	1	
POL	PRRSV-1-1_Poland_S351Vian	2011	MF600599	1	
POL	PRRSV-1-1_Poland_RJ1572-3	2014	MF600596	1	
POL	PRRSV-1-1_Poland_MPGB1-4	2014	MF600568	1	
POL	PRRSV-1-1_Poland_RJ1299-3	2014	MF600594	1	
POL	PRRSV-1-1_Poland_3143	2011	MF600547	1	1A
POL	PRRSV-1-1_Poland_17453-7	2010	MF600561	1	1B
POL	PRRSV-1-1_Poland_17451-8	2010	MF600558	1	1B
POL	PRRSV-1-1_Poland_17451-17	2010	MF600560	1	1B
POL	PRRSV-1-1_Poland_17451-14	2010	MF600559	1	1B
POL	PRRSV-1-1_Poland_17940-5	2010	MF600562	1	1B
POL	PRRSV-1-1_Poland_1111-10	2011	MF600544	1	1B
POL	PRRSV-1-1_Poland_MPGB2-10	2014	MF600582	1	1C
POL	PRRSV-1-1_Poland_MPGB2-19	2014	MF600585	1	1C
POL	PRRSV-1-1_Poland_MPGB2-27	2014	MF600587	1	1C
POL	PRRSV-1-1_Poland_MPGB2-4	2014	MF600579	1	1C
POL	PRRSV-1-1_Poland_MPGB2-5	2014	MF600580	1	1C
POL	PRRSV-1-1_Poland_MPGB2-25	2014	MF600586	1	1C
POL	PRRSV-1-1_Poland_MPGB2-15	2014	MF600584	1	1C
POL	PRRSV-1-1_Poland_MPGB2-7	2014	MF600581	1	1C
POL	PRRSV-1-1_Poland_MPGB2-11	2014	MF600583	1	1C
POL	PRRSV-1-1_Poland_MPGB2-29	2014	MF600588	1	1C
POL	PRRSV-1-1_Poland_RJ1180-9	2014	MF600593	1	1C
POL	AF378805_Bie94	1994		1	1D
POL	AF378810_Bie97	1997		1	1D
POL	AF378808_Zbr	1997		1	1D
POL	AF378816_Gro	2000		1	1D
POL	AF378818_Bie01	2001		1	1D
POL	AY641473_EU_NA	2002		1	1D
POL	PRRSV-1-1_Poland_Gol	2004	MF600565	1	1D
POL	DQ324680_Prz-71-75	2005		1	1D
POL	DQ324679_Prz-66-70	2005		1	1D

POL	JF730994_EU-12a_(CHE)	2005		1	1D
POL	DQ324673_Che-46	2005		1	1D
POL	DQ324688_Upa-13	2005		1	1D
POL	PRRSV-1-1_Poland_16410-8	2010	MF600557	1	1D
POL	PRRSV-1-1_Poland_16410-4	2010	MF600556	1	1D
POL	PRRSV-1-1_Poland_6346Wilc	2010	MF600554	1	1G
POL	PRRSV-1-1_Poland_RJ1546-1	2014	MF600595	1	1G
POL	PRRSV-1-1_Poland_RJ1834-20	2014	MF600597	1	1G
POL	AF378811_Rak	1997		3	
POL	AF378817_Krz	2000		3	
POL	DQ324675_Dzi-62	2005		3	
POL	PRRSV-1-1_Poland_17940-7	2010	MF600563	3	3D
POL	PRRSV-1-1_Poland_15824-2	2010	MF600555	3	3D
POL	PRRSV-1-1_Poland_17940-21	2010	MF600564	3	3D
POL	PRRSV-1-1_Poland_3191-5	2010	MF600550	3	3D
POL	PRRSV-1-1_Poland_3191-3	2010	MF600548	3	3D
POL	PRRSV-1-1_Poland_3191-4	2010	MF600549	3	3D
POL	PRRSV-1-1_Poland_946-26-30PK	2011	MF600537	3	3D
POL	PRRSV-1-1_Poland_946-6-10PK	2011	MF600536	3	3D
POL	PRRSV-1-1_Poland_495-5PK	2011	MF600535	3	3D
POL	PRRSV-1-1_Poland_S470Vian	2011	MF600601	3	3D
POL	PRRSV-1-1_Poland_MPGB1-15	2014	MF600575	3	3D
POL	PRRSV-1-1_Poland_MPGB1-14	2014	MF600574	3	3D
POL	PRRSV-1-1_Poland_MPGB1-26F	2014	MF600578	3	3D
POL	PRRSV-1-1_Poland_MPGB1-6	2014	MF600569	3	3D
POL	PRRSV-1-1_Poland_MPGB1-12	2014	MF600572	3	3D
POL	PRRSV-1-1_Poland_MPGB1-16	2014	MF600576	3	3D
POL	PRRSV-1-1_Poland_MPGB1-13	2014	MF600573	3	3D
POL	PRRSV-1-1_Poland_MPGB1-9	2014	MF600571	3	3D
POL	PRRSV-1-1_Poland_MPGB1-25	2014	MF600577	3	3D
POL	PRRSV-1-1_Poland_MPGB1-7	2014	MF600570	3	3D
POL	PRRSV-1-1_Poland_RJ740-1-3	2014	MF600592	3	3D
POL	PRRSV-1-1_Poland_RJ0739-15	2014	MF600591	3	3D
POL	PRRSV-1-1_Poland_RJ2085-1-4	2014	MF600598	3	3D
POL	PRRSV-1-1_Poland_RJ0431_1-4	2014	MF600589	3	3D
POL	PRRSV-1-1_Poland_RJ0431-5-8	2014	MF600590	3	3D
POL	AF378807_Kon	1996		3	3E
POL	AF378812_Nie	1997		3	3F
POL	AF378814_Jed	1998		3	3F
POL	DQ324684_Sok-4	2004		3	3F
POL	PRRSV-1-1_Poland_Kar	2009	MF600567	3	3F
POL	PRRSV-1-1_Poland_6346Seko	2010	MF600553	3	3F
ROU	JX099575_Rom17	2011		1	
ROU	JX099576_Rom18	2011		1	
ROU	JX099579_Rom19	2011		1	

ROU	JX099573_Rom15	2011		1	
ROU	JX099572_Rom14	2011		1	
ROU	JX105431_Rom21	2011		1	
ROU	JX099574_Rom16	2011		3	
ROU	JX090164_Rom9	2011		3	3A
ROU	JX075096_Rom5	2011		3	3A
ROU	JX090165_Rom10	2011		3	3A
ROU	JX090163_Rom7	2011		3	3A
ROU	JX090166_Rom11	2011		3	3A
ROU	JX090167_Rom8	2011		3	3A
ROU	JX075094_Rom3	2011		3	3A
ROU	JX099578_Rom13	2011		3	3A
ROU	JX075097_Rom6	2011		3	3A
ROU	JX099577_Rom12	2011		3	3A
SRB	PRRSV-1-1_Serbia_E14	2008	MF600613	3	3C
SRB	PRRSV-1-1_Serbia_E8	2008	MF600607	3	3C
SRB	PRRSV-1-1_Serbia_E5	2008	MF600604	3	3C
SRB	PRRSV-1-1_Serbia_E6	2009	MF600605	3	3C
SRB	PRRSV-1-1_Serbia_E9	2009	MF600608	3	3C
SRB	PRRSV-1-1_Serbia_E10	2009	MF600609	3	3C
SRB	PRRSV-1-1_Serbia_E13	2009	MF600612	3	3C
SRB	PRRSV-1-1_Serbia_E4	2009	MF600603	3	3C
SRB	PRRSV-1-1_Serbia_E7	2009	MF600606	3	3C
SRB	PRRSV-1-1_Serbia_E12	2010	MF600611	3	3C
SRB	PRRSV-1-1_Serbia_E15	2010	MF600614	3	3C
SRB	PRRSV-1-1_Serbia_E11	2010	MF600610	3	3C
SVK	PRRSV-1-1_Slovakia_Vos-17578	1996	MF600616	1	
SVK	KC522645_16M_SVK	2007		1	1G
SVK	KC522643_13M_SVK	2007		1	1G
SVK	PRRSV-1-1_Slovakia_Vos-2229	2007	MF600615	3	3F
SVK	KC522646_28M_SVK	2007		3	3F
SVK	KC522638_8M_SVK	2007		3	3F
SVK	KC522641_11M_SVK	2007		3	3F
SVK	KC522640_10M_SVK	2007		3	3F
SVN	PRRSV-1-1_Slovenia_1659	2012	MF600618	1	
SVN	PRRSV-1-1_Slovenia_975	2013	MF600617	1	
SVN	PRRSV-1-1_Slovenia_8167	2013	MF600622	1	
SVN	PRRSV-1-1_Slovenia_6770-2	2013	MF600621	1	
SVN	PRRSV-1-1_Slovenia_6405	2013	MF600620	1	1A
SVN	PRRSV-1-1_Slovenia_5852	2013	MF600619	3	3B
SVN	PRRSV-1-1_Slovenia_8220-3_20119-3R	2011	MF600623	3	3D
THA	AY297121_02BR1	2002		1	
THA	AY297122_02CB12	*2003		1	
THA	JQ040770_clone_AF0808EU_1	2010		1	
THA	JQ040768_clone_AF708EU_1	2010		1	

THA	KF698637_EU_TH_PLK048	2011		1	
THA	FJ908074_02SB3_na	2002		1	1F
THA	JQ040733_clone_AF04EU2.58-3	2010		1	1F
THA	JN002328_2PRRN2S10EU	2010		1	1F
THA	JQ040722_clone_AF08EU5.69-706	2010		1	1F
THA	JN002335_2AS10EU	2010		1	1F
THA	JX183113_SCP1210EU7_79-A07	2010		1	1F
THA	JQ040736_clone_AN06EU4.20-1	2010		1	1F
THA	JQ040735_clone_AN07EU4.52-5	2010		1	1F
THA	JQ040742_clone_AN08EU5.62-702	2010		1	1F
THA	JX183117_UD1210EU25_1	2010		1	1F
THA	JX183125_SCP0311EU3_1	2011		1	1F
THA	JX183123_SCP0311EU1_1	2011		1	1F
THA	JX183119_SCP0311EU1_2	2011		1	1F
USA	AY422801_MN-01-05_EU_2001_USA_MN	2001		1	
USA	AY395078_SD-02-11_USA_NE	2002		1	
USA	AF514803_NA_NA_NorA	*2002		1	
USA	EU758247_0000008116_2005_USA_UT	2005		1	
USA	EU758084_0000007860_2005_USA_UT	2005		1	
USA	EU758562_0000008510_USA_UT	2006		1	
USA	EU758589_0000008547_2006_USA_UT	2006		1	
USA	EU758891_0000008912_USA_IA	2007		1	
USA	EU759749_2000000593_2008_USA_NT	2008		1	
USA	EU758974_0000009020_2007_USA_VA	2007		1	1A
USA	DQ477805_0003791	*2006		1	1F
vaccine strain	DQ324678_Porcilis_PRRS			1	
vaccine strain	AF378819_Porcilis_PRRS			1	
vaccine strain	GU067771_Amervac_PRRS			1	
vaccine strain	AF378820_Pyrsvac-183_ESP			1	
vaccine strain	DQ324681_Pyrsvac-187_ESP			1	
vaccine strain	DQ064787_V3_ESP			1	
